# An Overview of Cardiovascular Risk in Pituitary Disorders

**DOI:** 10.3390/medicina60081241

**Published:** 2024-07-30

**Authors:** Georgia Ntali, Vyron Markussis, Alexandra Chrisoulidou

**Affiliations:** 1 Department of Endocrinology “D. Ikkos”, Diabetes Center, Evangelismos General Hospital, 10676 Athens, Greece; 2 Independent Researcher, 18535 Athens, Greece; vyron13@otenet.gr; 3 Department of Endocrinology, Theagenio Cancer Hospital, 54636 Thessaloniki, Greece; a.chrisoulidou@gmail.com

**Keywords:** Cushing’s Disease, acromegaly, prolactinoma, hypopituitarism, cardiovascular morbidity, mortality

## Abstract

Cardiovascular comorbidities owing to hormonal excess or deficiency are the main cause of mortality in patients with pituitary disorders. In patients with Cushing’s Disease, there is an increased prevalence of cardiovascular diseases and/or risk factors including visceral obesity, insulin resistance, atherosclerosis, arterial hypertension, dyslipidaemia, hypercoagulability as well as structural and functional changes in the heart, like cardiac hypertrophy and left ventricle (LV) dysfunction. Notably, these demonstrate limited reversibility even after remission. Furthermore, patients with acromegaly may manifest insulin resistance but also structural and functional heart changes, also known as “acromegalic cardiomyopathy”. Patients with prolactinomas demonstrate an aggravation of metabolic parameters, obesity, dysregulation of glucose and lipid metabolism as well as endothelial dysfunction. Hypopituitarism and conventional hormonal replacement therapy may also contribute to an unhealthy metabolic status, which promotes atherosclerosis and may lead to premature mortality. This review discusses the literature on cardiovascular risk in patients with pituitary disorders to increase physician awareness regarding this aspect of management in patients with pituitary disorders.

## 1. Introduction

The pituitary gland plays a pivotal role in the regulation of metabolism as well as the physiologic function of the cardiovascular (CV) system. These effects are mediated through endocrine signals generated by secreted pituitary hormones. Glucocorticoids (GCs) increase caloric and dietary fat intake, induce hyperglycaemia and ectopic fat distribution, while also acting directly on the heart and blood vessels by promoting hypertension and atherosclerosis. Patients with Cushing’s Disease (CD) typically manifest visceral obesity, insulin resistance, atherosclerosis, arterial hypertension, dyslipidaemia, hypercoagulability as well as structural and functional changes in the heart, in the form of cardiac hypertrophy and left ventricle (LV) dysfunction. Growth hormone (GH) promotes insulin resistance and lipolysis and regulates cardiac contractility and vascular motility. Acromegaly may result in glucose and lipid abnormalities and various structural and functional heart disorders, collectively characterized as acromegalic cardiomyopathy. Prolactin regulates food intake and exerts inotropic effects on the myocardium. Patients with prolactinomas frequently have endothelial dysfunction and even abnormalities of systolic and diastolic heart function. Hormonal excess or deficiency caused by pituitary adenomas can potentially lead to several secondary comorbidities. However, the development of CV diseases constitutes the main cause of mortality in these patients [1,2,3,4,5,6,7,8,9,10,11,12]. Various treatment modalities (surgery, medical treatment or radiotherapy) of pituitary adenomas may also affect the cardiometabolic status of the patient (Table 1).

**Figure 1:**
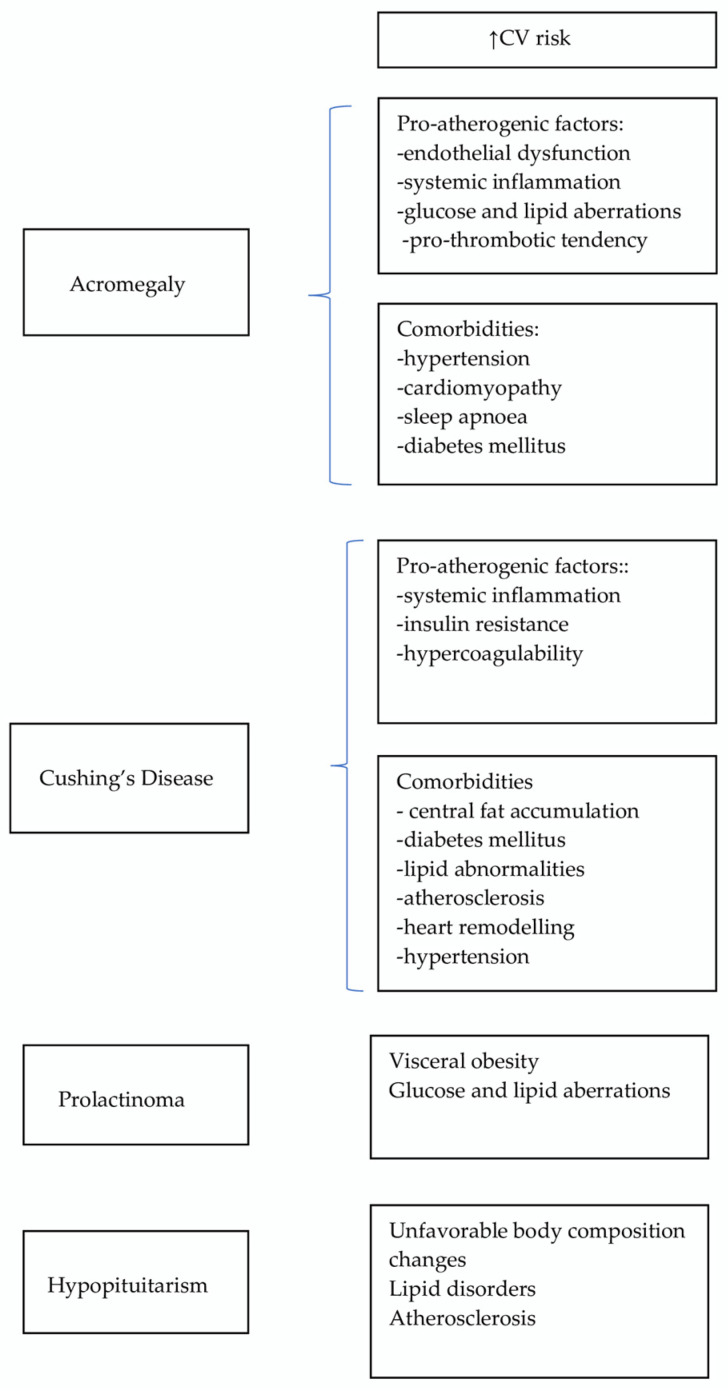
CV risk factors in pituitary disorders—key points.

Herein, we review the literature on CV risk in patients with CD, acromegaly, prolactinomas and hypopituitarism and we report new findings in the area (Table 2) and a schematic format in Figure 1 of the mechanisms behind the increased CV risk. 

## 2. Cushing’s Disease

### 2.1. Mortality

CD is a rare condition of cortisol excess attributed to an adrenocorticotropic hormone (ACTH)-secreting adenoma. Chronic hypercortisolaemia is associated with CV abnormalities, which do not reverse completely even after remission [2,3,4,5,6,7,8,9]. The incidence of cardiac and cerebrovascular comorbidities was investigated in 503 patients with CD during the period before diagnosis, from diagnosis to 1 year after remission, and during long-term remission. A three-fold increased standard incidence ratio (SIR) was observed for stroke, two-fold for myocardial infarction, five-fold for pulmonary embolism and three-fold for deep vein thrombosis. SIR for thromboembolism was increased in both the period before diagnosis (11.5, 95% CI 4.2–25.0) and the peri- treatment period (18.3, 95% CI 7.9–36) and remained elevated during long term remission for both stroke (3.1, 95% CI 1.8–4.9) and thromboembolism (4.9, 95% CI 2.6–8.4) [1]. 

CV disease is the main cause of increased mortality which is observed not only in patients with persistent hypercortisolaemia but also in those in remission [2,3,4,5,6,7,8,9]. A metanalysis of 13 CD cohorts reported that pooled Standard Mortality Ratio (SMR) for CD was 2.8 (95% CI, 2.1–3.7), with particularly elevated risk in patients with active CD (SMR 5.7) and lower but still elevated risk in those in remission (SMR 2.3, *p* < 0.001) [13]. These data highlight the irreversible negative effect of chronic hypercortisolaemia on the CV and cerebrovascular system [8].

### 2.2. Metabolism

#### 2.2.1. Obesity

GCs increase appetite and lead to high caloric intake. In adipose tissue, GCs increase adipogenesis and triglyceride synthesis, as well as lipid uptake and storage. The accumulation of fatty acids in the circulation results in ectopic fat distribution in the liver, muscle, and central adipocytes. In vitro experiments show that high cortisol increases lipolysis in the presence of insulin in subcutaneous, but not visceral, adipocytes which may explain the fat accumulation in state of cortisol excess [14].

Chronic hypercortisolaemia can adversely affect body composition. It increases the total and trunk fat and reduces the lean body mass [15]. A study employing magnetic resonance imaging (MRI) showed that females with active CD had significantly higher total, visceral and trunk subcutaneous adipose tissue, but similar intermuscular fat, despite lower skeletal mass, compared to weight matched controls [15]. Prolonged exposure to GCs suppresses the function of brown adipose tissue (BAT) and favors energy production and lipogenesis [16]. Body composition changes remain impaired despite long-term remission. In particular, when patients were studied 5 years after remission, they still had significantly higher body mass index (BMI) and waist-to-hip ratio (WHR) in comparison with age-, sex- and BMI-matched controls [17]. Women with CS in long-term remission have increased abdominal fat mass compared with healthy subjects, which is independently associated with GC replacement and a common polymorphism in the GC transmembrane transporter gene ABCB1 [18]. The adipose tissue of patients with active CD demonstrates an increased infiltration by CD68+ macrophages, CD4+ T lymphocytes, and CD11c+ macrophages, as well as decreased vimentin levels compared with BMI-matched controls [19]. Furthermore, an altered adipokine profile characterized by lower fetuin A levels, higher fatty acid binding protein (FABP4) and retinol binding protein (RBP4) concentrations have been measured in CS compared to controls [20].

Remission of CD for 6 months improves fat distribution and ameliorates but does not completely restore the metabolic profile [21]. IL-6 and IL-1β levels remained significantly elevated in a group of 31 patients with CD despite their remission and subsequent reduction of BMI, insulin resistance and visceral, hepatic and intermuscular fat stores [22]. Tumor Necrosis Factor-α (TNF-α) remains elevated in patients cured from CS and is associated with the presence of coronary calcification. The status of low-grade inflammation, contributes to the increased CV mortality observed amongst patients with CD [23]. Excessive total and trunk fat and the inflammatory profile with low adiponectin and high TNF-α and IL-6 may persist even 11 years after remission of CD [24].

#### 2.2.2. Glucose Metabolism

In the liver, GCs upregulate gluconeogenesis and glycogenolysis while at the level of muscle and adipose tissue they inhibit peripheral glucose uptake resulting in hyperglycemia. In the pancreas, cortisol decreases insulin and increases glucagon secretion, which enhances liver ketogenesis and lipolysis and decrease lipogenesis [25]. Together with insulin they promote non-esterified fatty acid (NEFA) uptake by hepatocytes and triglyceride synthesis, which promotes hepatic steatosis. 

Amongst 25 patients with CD and 32 sex- and age-matched subjects (control-1 group) and 32 BMI-matched subjects (control-2), the prevalence of impaired glucose metabolism was higher in patients with CD and recovered only in two patients after 1 year in remission [26]. Furthermore, patients with CD in remission for 5 years had higher fasting and stimulated glucose and insulin levels than sex- and age-matched controls. Diabetes mellitus was diagnosed in five patients and in two BMI- matched controls, whereas reduced glucose tolerance was found in four patients, in three sex- and age-matched controls, and in eight BMI-matched controls [17].

#### 2.2.3. Lipid Metabolism

Excess GCs cause dyslipidaemia due to inhibition of lipoprotein lipase (LPL) activity in adipose tissue. Patients with active CD have higher total cholesterol, low density lipoprotein-cholesterol (LDL), total/high-density lipoprotein (HDL) ratio and lower HDL levels than sex- and age-matched subjects and higher total/HDL ratio and lower HDL levels than BMI matched subjects [27]. One year post remission total and LDL cholesterol were shown to decrease [26,28].

### 2.3. Cardiovascular System

#### 2.3.1. Hypertension

GCs affect angiogenesis, oxidative stress and inflammation in vascular smooth muscle and endothelial cells, while also exerting their actions through glucocorticoid and mineralocorticoid receptors on heart and blood vessels. A well-recognized effect is the inhibition of nitric oxide biosynthesis [29]. 

Approximately 80% of patients with CD suffer from hypertension [30]. The underlying pathogenesis includes the activation of mineralocorticoid and glucocorticoid receptors, the activation of the renin-angiotensin axis and sympathetic nervous system and an imbalance between substances favoring vasodilatation and vasoconstriction [31,32]. Electrocardiographic abnormalities with longer QTcd (QTc dispersion) and shorter QTcmin (QT corrected for heart rate min), left and right ventricular hypertrophy (LVH/RVH) and higher systolic and diastolic blood pressure are common findings [30]. The Ambulatory Arterial Stiffness Index (AASI) and blood pressure variability are increased in patients with CS, independent of blood pressure [33,34]. Approximately 50% of patients with active CD lack nocturnal blood pressure dips, which remain absent even 1 year after remission. Daytime heart rate is higher in patients with active CD and decreases over time after curative treatment [35]. In a group of 29 patients with CS (14 with CD), 64% of patients with active disease were hypertensive but 50% still required antihypertensive medication 1 year after remission [36]. 

#### 2.3.2. Heart and Vessels

Increased intima-media thickness (IMT) of carotid and aorta, lower systolic lumen diameter and atherosclerotic plaques have been detected [27]. Capillary microarchitectural changes are frequent [37]. Higher levels of epicardial adipose tissue and coronary plaque have been reported using coronary computed tomography angiography in patients with Cushing Syndrome (CS) in comparison with controls. The duration and severity of hypercortisolaemia are prognostic factors [38].

A metanalysis of 14 studies (332 CS, 462 controls) estimated that compared with controls, CS patients showed higher IMT, increased prevalence of carotid plaques and lower flow mediated dilation (FMD) and these differences remained in patients in remission [39]. One year post remission, LDL-cholesterol and IMT were significantly decreased and systolic lumen diameter was significantly increased, similarly to BMI matched subjects but still abnormal compared with sex and age matched controls [26]. Coronary calcifications and/or noncalcified plaques have been demonstrated in 42% of women and 30% of patients (<45 yrs) with remission for 11 years [22]. Echo doppler ultrasonography has detected impaired atheromatic indices after remission of 5 years [17]. In contrast, another study that compared vascular markers and endothelial function between patients with CS in remission ≥ 4 years and controls reported no significant differences [40].

In patients with active CD, impaired diastolic and systolic LV function has been reported [32,41]. Approximately 70% of patients manifest abnormal LV mass parameters. Concentric hypertrophy has been described in 42% and concentric remodelling in 23% [42]. Hypercortisolaemia induces an early remodelling of left and right ventricles, independent of traditional cardiometabolic risk factors, 24 h BP load and profile (dipping/non-dipping), which persists after CS remission [26,43,44]. Patients with CD exhibit a greater degree of vasoconstriction and LV systolic dysfunction in comparison with hypertensive middle-aged controls. Even those with well-controlled blood pressure (below 140/90 mmHg) demonstrate significantly lower LV contractility and higher prevalence of LV diastolic dysfunction in comparison with hypertensive patients and healthy volunteers [45]. 

Cortisol-related changes in myocardial content have been detected using MRI, both at active state and after treatment. Native myocardial T1 relaxation time is increased in patients with CD compared with matched healthy and hypertensive individuals and decreases after effective treatment [46]. Obesity also negatively affects cardiac function and impedance cardiography has detected differences between obese and non-obese patient with CD [47]. Subsequently, patients in long-term remission from CS continue to have a lower aerobic exercise capacity compared with a well-matched, healthy control group [48].

### 2.4. Thrombotic Risk

Hypercortisolism provokes a hypercoagulability state predisposing to venous and arterial thromboembolic events [49]. The underlying mechanisms include (i) increase in pro-coagulation factors and shortened activated partial thromboplastin time (aPTT), (ii) impaired fibrinolysis, (iii) increased thrombin, thromboxane A2 and platelets, and (iv) compensatory increase in anticoagulation factors such as protein C and S. Moreover, endothelial dysfunction, increased IMT, vascular wall fibrosis and remodelling, increased vascular oxidative stress and insulin resistance contribute to haemostatic imbalance. Haemoglobin and haematocrit values are higher in patients with CS, and normalize after remission [50]. Sexual and subtype-specific differences in erythrocyte parameters have been reported and women with CS show higher haematocrit/haemoglobin levels, than men [51].

The risk for venous thromboembolic events (VTE) after pituitary surgery is higher in patients with CD (3.4%) compared with patients with non-functioning pituitary adenomas (NFAs) (0%). The incidence rate for patients with CD was 141 (95% CI 75–234) per 1000 person-years and the majority of events occurred between 1 week and 2 months after pituitary surgery. Medically pre-treated patients had a lower risk of VTE in the 3 months after surgery (2.5%, 95% CI 1.2–5.1) compared with those who were not pre-treated (7.2%, 95% CI 3.1–15.9) [52]. A metanalysis of 2083 cases undergoing either transsphenoidal (TSS) surgery (1476 cases) or adrenalectomy (607 cases) estimated that 4.75% (99/2083) of patients had a VTE event within 30 days of surgery [53]. A possible explanation is that postoperative reduction of cortisol activates inflammation and increases coagulation parameters and these abnormalities persist up to 1 year or longer after remission. Thrombogenic and proinflammatory factors have been detected in the circulation of premenopausal women with CS in remission who had no evidence of atheromatosis [54]. Recent guidelines emphasize that screening for CV risk factors and rigorous management should be performed during all stages of management of CD [55].

### 2.5. Effects of Specific Treatments on Metabolic and Cardiovascular Issues

Treatment with levoketoconazole improves glycaemic control in diabetic patients [56]. Long term treatment with osilodrostat ameliorates metabolic and cardiovascular parameters and decreases weight, BMI, waist circumference, blood pressure, fasting plasma glucose, HbA1c, total cholesterol, and triglycerides [57]. Pasireotide therapy significantly reduces weight, BMI, waist circumference, as well as total and LDL-cholesterol, while significantly increases fasting plasma glucose and glycated haemoglobin [58,59]. Mifepristone is the only FDA-approved drug for glycaemic control in patients with CS and type 2 diabetes. It enhances insulin-stimulated glucose uptake through a mechanism that involves a decrease in mitochondrial function and AMPK activation in skeletal muscle cells [60].

## 3. Acromegaly

### 3.1. Mortality

Acromegaly is characterized by increased levels of growth hormone (GH) and insulin-like growth factor-1 (IGF-1) and is caused in the majority of cases by a GH-producing pituitary adenoma. Older studies reported increased mortality by two-three times, mainly due to CV and respiratory causes [61]. However, recent reports describe a reduction in SMR to levels similar to the general population with malignancies being the main cause of death [62,63,64]. SMR of acromegalic patients in Sweden was 1.29 (95% CI 1.11–1.49). It was not increased in controlled acromegalics but it was elevated in non-controlled patients at the latest follow-up (1.90, 95% CI 1.33–2.72) (for the decade 1991–2000) and 1.98 (95% CI 1.24–3.14) (for the decade 2001–2011), respectively [65]. Both increased GH levels and duration of disease affect CV risk. An average delay for diagnosis and initiation of treatment of about 10 years has been estimated. The adoption of current criteria of remission, safe GH levels (random < 1 ng/mL, nadir during oral glucose tolerance test < 0.4 ng/mL) and age/gender normalized IGF-1 levels together with modern therapeutic modalities have improved patient outcomes [66]. Data from the UK Acromegaly Registry Data showed an increase in all-cause mortality (SMR, 1.35; 95% CI: 1.24–1.46, *p* < 0.001), mostly attributed to CV disease (SMR 1.38; 95% CI: 1.16–1.63, *p* < 0.001), cerebrovascular disease (SMR 1.49; 95% CI: 1.10–1.97, *p* = 0.006) and respiratory disease (SMR 1.55; 95% CI: 1.22–1.93, *p* < 0.001). CV and cerebrovascular mortality rates and all-cause mortality were positively correlated with post-treatment GH levels. With post-treatment GH levels of <2.5 µg/L (SMR 1.15; 95% CI: 1.03–1.28, *p* < 0.001) all-cause mortality was reaching those of normal population [10]. In the German Acromegaly Registry, SMR for myocardial infarction was 0.89, and for stroke 1.17 [67].

### 3.2. Metabolism 

#### 3.2.1. Body Composition

The metabolic effects of GH predominantly involve the stimulation of lipolysis in the adipose tissue resulting in an increased flux of free fatty acids (FFAs) into the circulation. In the muscle and liver, GH stimulates triglyceride (TG) uptake, by enhancing lipoprotein lipase (LPL) expression, and its subsequent storage.

No differences have been detected in body composition between controlled and not controlled acromegalics by the means of bioimpedance or dual-energy X-ray absorptiometry (DEXA) analysis [68,69]. Interestingly, a type of lipodystrophy with reduced adipose mass centrally and intrahepatic lipid and a shift of excess lipid to intramuscular adipose tissue has been observed [69]. The increased adipose content in muscle could be associated with GH-induced insulin-resistance [70]. Visceral adipose tissue (VAT) is positively associated with glucose and lipid metabolism and negatively with GH [71]. After surgical treatment of acromegaly VAT, subcutaneous adipose tissue (SAT), and intrahepatic lipid content (IHL) gains persist long-term and SAT depot is associated with improvement in insulin resistance possibly because of better lipid distribution [72].

GH directly promotes inflammation of human adipocytes by increasing Vascular Endothelial Growth Factor (VEGF) and Monocyte Chemoattractant Protein 1 (MCP1) levels [73] and the expression of genes encoding adipokines, such as visfatin and IL-6. Therefore, although patients with acromegaly exhibit a reduced fat mass compared with healthy subjects, the adipose tissue can display an inflammatory phenotype that contributes to the elevated lipid levels and insulin resistance.

#### 3.2.2. Glucose Metabolism

The role of GH and IGF-1 in glucose metabolism is complex. GH directly promotes an insulin resistant state as it antagonizes insulin action and increases hepatic gluconeogenesis [74]. In addition, it stimulates TG secretion and hepatic fatty acid oxidation. Glucose metabolism dysregulation is a frequent complication of acromegaly and further contributes to the increased CV risk and mortality. 

The prevalence of abnormal glucose tolerance is more than 50% at the time of diagnosis and it is positively associated with older age, higher BMI, family history of diabetes and higher IGF-1 z-score, but not with fasting or post-OGTT GH levels [75]. The negative effect of diabetes on the CV mortality of acromegalics was clearly demonstrated in the Swedish, nationwide study which compared 254 acromegalics with type 2 diabetes (ACRO-DM group) with 532 acromegalics without diabetes (ACRO group) for a mean follow-up of 9.2 years. The unadjusted overall mortality rate per 1000 person-years was 35.1, (95% CI, 27.2–44.7) and 20.1, (95% CI, 16.5–24.3), respectively. The Hazard Ratio (HR) for overall mortality was 1.58, (95% CI, 1.12–2.23), while the HR for CV mortality was 2.11, (95% CI, 1.09–4.10), for CV morbidity 1.49, (95% CI, 1.21–1.82) and were increased in the ACRO-DM group [76].

Acromegalic patients have insulin resistance and hyperinsulinemia, but lower IHL content compared with age-, BMI-, and sex-matched healthy controls [77]. Patients with diabetes exhibit hyperglucagonemia and compromised β-cell function despite significantly higher glucose dependent insulinotropic polypeptide (GIP) levels. After surgery, indices of insulin sensitivity improve, GIP and glucagon levels decrease significantly in both groups, but there is no significant change of β cell function those with hyperglycaemia [78]. Insulin resistance is further enhanced by dyslipidaemia and obstructive sleep apnea syndrome (OSAS), which are common disorders in acromegaly. Biochemical control of acromegaly improves insulin resistance [79] but leads to a less favorable anthropometric phenotype with increased IHL and abdominal adiposity and reduced muscle mass [77].

#### 3.2.3. Lipid Metabolism

The increased availability of FFAs promote insulin resistance, increases TGs and reduce HDL levels. GH inhibits lipoprotein lipase (LPL), which hydrolyzes TGs in the circulation through an FFA-related manner and upregulation of angiopoietin-like protein 4 (ANGPTL4) [80]. Acromegalic patients may manifest lower HDL cholesterol and apolipoprotein A1 (apo A1) levels and higher TGs and Lp(a) concentrations compared with controls [81,82]. HDL decline is associated with a reduction of lecithin cholesterol acyltransferase (LCAT), cholesteryl ester transfer protein (CETP) and phospholipid transfer protein (PLTP), which normally regulate HDL metabolism. An independent negative relationship with plasma IGF-1 levels has also been described [83]. The atherogenic profile is further enhanced by diminished antioxidant capacity due to decreased nitric oxide levels and increased oxidative stress markers [84]. Control of acromegaly is followed by reduction of Lp(a) and increase in apo A1 levels [71].

### 3.3. Cardiovascular System 

Under physiologic conditions the GH /IGF-I axis exerts a beneficial effect on the CV system. GH and IGF-1 receptors are expressed in the heart and the vessels and regulate myocyte growth and structure, cardiac contractility and vascular function [85,86]. 

#### 3.3.1. Hypertension

Hypertension is a major factor for CV mortality in acromegaly. Excess GH leads to increased sodium and water retention, by a direct kidney effect at the epithelial sodium channel (ENaC) subunit in the cortical collecting ducts. The prevalence is estimated at approximately 30%, while 50% of patients are estimated to be non-dippers. These changes are not always reversible and hypertension persists in many treated acromegalic patients [87,88]. The prevalence of hypertension in the ACROSTUDY cohort was 56% at study entry and increased to 64% during the follow-up period, with 182 newly diagnosed patients with hypertension during the study [89].

#### 3.3.2. Heart and Vessels

Acromegaly is characterized by systemic inflammation with high levels of vascular cell adhesion molecules (VCAM), E-selectin, Matrix Metalloproteinase (MMP)2, mostly in uncontrolled patents. Treatment only partially reverses these alterations [90]. In physiologic conditions, the PI3K-Act pathway reduces vascular tone and oxidative damage. Activation of different signaling pathways, including the activator of transcription 3 (STAT3), nuclear factor kappa B (NF-κB), nod-like receptor pyrin 3 (NLRP3) inflammasome, and mitogen-activated protein kinase (MAPK) induce systemic inflammation, endothelial dysfunction and vascular remodelling [90,91,92].

Increased endothelial proliferation, dysfunction of endothelial progenitor cells, increased oxidative stress and reduced levels of nitric oxide and eNOS expression have been reported in patients with active acromegaly [93]. eNOS expression and nitric oxide levels are inversely correlated with GH/IGF-1 levels [94]. Higher oxLDL levels have been reported, even after adjusting for glucose and insulin levels [95].

Pre-clinical markers of atherosclerosis such as arterial IMT, pulse wave velocity and FMD are affected [93]. Significantly lower FMD of the brachial artery has been detected in acromegalics than in healthy and risk-factor-matched controls [96]. A comparison of carotid IMT by ultrasonography showed a significant increase in acromegalic patients in one study [97] and no difference in another [93]. In asymptomatic acromegalic patients, coronary flow reserve (a marker of microvascular coronary function) was impaired and was partially reversed after treatment [98]. Approximately 40% of acromegalic patients were found to have evidence of coronary atherosclerosis and the control of acromegaly did not influence significantly the extent of coronary atherosclerosis [99]. 

GH can stimulate cardiomyocyte hypertrophy independent of IGF-1 [86]. IGF-1 has a direct hypertrophic effect on cultured rat cardiomyocytes through its own receptor leading to increased actin/myosin expression, increased intracellular calcium levels and an anti-apoptotic effect [92]. Acromegaly is associated with a typical cardiomyopathy, characterized by biventricular hypertrophy, myocardial necrosis, lymphocytic infiltration, and interstitial fibrosis [100]. Cardiac hypertrophy presents as a hyperkinetic syndrome with increased systolic output. As it worsens, diastolic dysfunction arises in up to 58% of patients with active disease, and systolic dysfunction appears on effort. The end-stage is characterized by systolic dysfunction at rest and overt congestive heart failure (CHF). The progression to systolic dysfunction is generally uncommon (<3%) and CHF is rare in active acromegaly (1–4%). The 1- and 5-year mortality (or transplantation) rates for patients with chronic symptomatic CHF are 25% and 37.5%, respectively, as dilated cardiomyopathy is not reversible. In echocardiography studies, 11–78% of acromegalic patients have LV hypertrophy and 11–58% have diastolic dysfunction. Dos Santos Silva et al. reported a lower prevalence of LVH (5%) with cardiac magnetic resonance (CMR) imaging than echocardiography and development of myocardial fibrosis in 14.8% of patients with acromegaly without significant difference between active and controlled disease [101]. In contrast, de Alcubierre et al. reported biventricular structural and functional impairment (LV-end-diastolic volume, LV-end-systolic volume, LV-mass, LV-stroke volume, RV-ejection fraction) in active and controlled acromegaly with CMR imaging, especially in men [102].

Cardiac arrhythmias and sudden cardiac death are the most common causes of increased mortality in acromegaly. There are reports of elevated beat-to-beat QT variability and occurrence of late potentials [103]. LV dyssynchrony is increased in acromegaly and may contribute to the progression to dilated cardiomyopathy [104]. Interstitial fibrosis is a potential contributing factor for arrhythmias. Valvulopathy has been reported, including mitral valve (5% vs. 0% in controls) and aortic valve regurgitation (20% vs. 4% in controls) with risk increasing by 19% per year with disease duration [105]. In a prospective study, mitral valve regurgitation increased from 46% to 76% in 2 years [106]. A recent echocardiography study reported valve defects in 87.3% of patients (14.6% with significant valvular heart disease) [107].

### 3.4. Thrombotic Risk

Abnormalities of haemostatic function include elevated levels of fibrinogen, antithrombin III, plasminogen activator Inhibitor-1 (PAI-1), enhanced platelet activity and reduced levels of protein C, S and t-PA [108,109,110]. Moreover, patients with active acromegaly have disturbed fibrin networks with more compact clots [111].

### 3.5. Other Cardiovascular Risk Markers in Acromegaly

#### 3.5.1. Phosphate Levels

Serum phosphate level has been suggested as a marker of acromegaly activity. It correlates with IGF-1 levels and the SAGIT acromegaly score and can be used to predict remission of the disease [112]. Phosphorus levels < 3.98 mg/dL and serum calcium levels < 9.88 mg/dL are predictors of acromegaly remission. Increased phosphorus may be linked with cardiovascular risk through impaired endothelial function. It directly induces vascular calcification via the activation of TLR4/NF-κB signaling in valvular smooth muscle cells [113].

#### 3.5.2. Ceruloplasmin

Ceruloplasmin activity, another risk factor for CV disease by contributing to LDL oxidation, is increased in acromegaly [95].

#### 3.5.3. sKlotho

The klotho gene encodes a transmembrane protein (mKL) expressed mainly in the kidneys and the soluble part, soluble alpha klotho (sKL), has been investigated as potential new marker in acromegaly. GH hypersecretion leads to increased IGF-1 and soluble Klotho levels. It has been proposed that sKL may be a biomarker of disease activity, as it is closely associated with GH secretory status, particularly useful in cases of GH and IGF-1 discrepancy. While mKL is a well characterized co-receptor of FGF23, preliminary data suggest that sKL may modulate downstream insulin signaling [114]. There are no available data for the use of sKL as a CV risk factor in acromegaly. Both GH and soluble Klotho induce insulin resistance via ectopic intramuscular adipose tissue deposition and adipose tissue inflammation.

#### 3.5.4. Molecular Markers

Several molecular markers have been studied in acromegaly like E-cadherin, ZAC-1, and aryl hydrocarbon receptor-interacting protein (AIP). However, they are used as diagnostic tools and for prediction of response to medical treatment, and have not been linked to CV outcomes [115].

### 3.6. Effects of Specific Treatments on Metabolic and Cardiovascular Issues

Despite the fact that mortality risk in acromegaly practically normalizes with adequate treatment, CV risk often persists [116]. Markers of inflammation and endothelial dysfunction detected through measurements of flow-mediated dilatation/nitroglycerine-mediated dilatation ratio remain impaired in cured acromegaly patients [117].

The surgical treatment of acromegaly has a beneficial effect on insulin resistance and glucose metabolism [87], however, patients with already compromised β-cell function do not improve after surgery [78]. A metanalysis of trials reported that neurosurgery improves metabolism with a significant decrease in fasting blood glucose (FPG), glucose load, HbA1c, fasting plasma insulin, Homeostasis Model Assessment Insulin Resistance (HOMA-IR), TGs, and LDL cholesterol and increase in HDL cholesterol [118].

Somatostatin analogues (SSAs) improve insulin sensitivity, but on the other hand inhibit insulin secretion [119]. In a prospective study, the use of SSAs as primary medical treatment for 5 years improved dyslipidaemia, hypertension and cardiac performance while glucose tolerance remained stable [120]. A recent meta-analysis showed that SSAs improve disease control, reduce insulin levels, and increase HbA1c levels without affecting FPG [121]. Glucose monitoring is necessary during treatment with SSAs, particularly for the second generation SSA pasireotide. Approximately 25% of patients who received pasireotide (responders and non-responders) developed diabetes [122]. A metanalysis of 13 interventional studies showed that the GH antagonist pegvisomant, in monotherapy or combined with SSAs improves glucose metabolism, reducing FPG, HbA1c, FPI and HOMA-IR [123]. Both surgery and SSAs have been reported to reduce TGs and increase HDL levels in acromegalic patients [120,124]. Pegvisomant significantly increases LDL and total cholesterol, whereas SSAs have no effect on LDL levels [125]. Cabergoline improves BMI in acromegaly but not glucose metabolism [126].

Hypertension responds to amiloride [88], and ACE-i or ARBs improve cardiac indices in CMR compared with other antihypertensive drugs [127].

In a prospective study where patients received 6 months SSAs, IMT tended to decrease only in patients with disease control [128]. Similarly, in another study a slight reduction of carotid arteries wall thickness and a significant improvement of brachial artery vascular function was observed in patients with acromegaly resistant to SSA who were treated with pegvisomant [129]. cIMT and pulse wave velocity (PWV), which are markers of atherosclerosis are higher in the acromegaly group than in the healthy group and the fact that they do not differ between active and controlled acromegaly shows that defects may be irreversible [130].

CV disease improves after acromegaly treatment by either surgery, SSA or pegvisomant. In a prospective study, many cardiac indices (LVM, LVMi, interventricular septum diastolic thickness (IVSDT) and posterior wall diastolic thickness (PWDT)) were significantly reduced and diastolic function was significantly improved within 6 months after surgery [131]. The reversibility of LVH by SSAs appears by 3 months, and cardiac remodelling occurs quite quickly [132]. Reduction of LV mass correlates with the response to SSAs treatment [133]. SSAs have direct beneficial effects on cardiac myocytes and contribute to the improvement of echocardiographic parameters even in patients who have not achieved complete biochemical control of the disease. Diastolic filling is improved, but the effect on ejection fraction and exercise tolerance is more variable. They have beneficial effects on cardiomyopathy that are not apparent in surgically treated patients. The recovery of LV hypertrophy or diastolic and systolic dysfunction depends not only on the correction of hormone excess but also on patient age and disease duration [134]. In patients not controlled on SSAs alone, pegvisomant monotherapy [135] or in combination with SSAs [136] improves acromegalic cardiomyopathy.

In a cohort of acromegalic patients who presented with arrhythmias, a significant amelioration was observed after 6 months treatment with SSAs [137]. A risk of QT interval prolongation has been reported with pasireotide [138]. Some studies have shown no change in the frequency of ventricular arrhythmias after successful treatment of acromegaly, possibly due to the irreversibility of myocardial fibrosis in some cases. In contrast, acromegalic patients without structural heart disease had a low frequency of cardiac arrhythmias [139]. Pegvisomant has also been reported to improve arrhythmias [140]. Approximately 11–30% of patients with acromegaly suffer from OSAS which, despite cure of acromegaly, persists in more than 40% of patients in prospective studies [141].

Of note, the incidence of valvular abnormalities and the risk for further progression of valvulopathy remain unchanged with treatment. Fortunately, in patients with acromegaly treated with cabergoline, data are reassuring regarding the risk for cardiac valve disease [142].

Coagulation and fibrinolysis aberrations may resolve after surgical or medical treatment. Fibrinogen levels are reduced in controlled patients [128] and platelet volume and function are at least partially normalized [143]. Similarly, the abnormal clot structure properties in active acromegaly seem to ameliorate in patients with long term disease remission [111].

Real world data show that comorbidities are less prevalent in controlled patients, but unfortunately a third of the patients remained uncontrolled after 8 years of treatment which demonstrates the difficulty of achieving control in some patients [144].

The exact prevalence of GH deficiency (GHD) after acromegaly treatment is 10% to 50% after pituitary surgery and 30–75% after radiation. Overtreatment of acromegaly can be avoided in patients receiving medical treatment [145]. The presence of GHD is linked to high cardiometabolic risk. Regarding IGF-1, a U-shaped relationship was demonstrated for cardiovascular mortality in a meta-analysis and data were suggestive of optimal IGF-I levels between 0 and +1 SD in adult patients [146]. In this context, low-dose GH replacement has been tried, but long-term cardiovascular risk remains to be investigated.

## 4. Prolactinomas

### 4.1. Mortality

CVD related mortality is lower in prolactinomas compared with patients diagnosed with ACTH- or TSH-secreting adenomas or NFAs. SIR for hemorrhagic stroke was 3.88, for ischemic stroke 2.94, and for acute myocardial infarct 1.94 in a study from Korea [147]. Cardiovascular incidence rate is higher in males (14.8 per 1000 person-years versus 1.8) for the females [11].

### 4.2. Metabolism

#### 4.2.1. Obesity

Prolactin excess influences the orexigenic–anorexigenic systems and results in increased food intake, leading to weight gain and obesity [148].

Patients with prolactinomas and particularly men have an increased prevalence of obesity when compared with the general population [149,150,151]. Normalization of prolactin with either TSS or dopamine agonists (DAs) is associated with BMI reduction [152,153].

#### 4.2.2. Glucose Metabolism

Prolactin and type 2 dopamine receptors are expressed on both human pancreatic β-cells and adipocytes, supporting a key role of prolactin and dopamine in peripheral metabolic regulation. Data for glucose metabolism are not consistent and either higher fasting glucose levels [83] or similar to controls [150] have been observed. DAs improve insulin sensitivity [153,154] independent of prolactin reduction [155]. Reduction of fasting glucose has been observed after normalization of prolactin with either TSS or DA therapy [156].

#### 4.2.3. Lipid Metabolism

Patients with prolactinoma have higher levels of LDL and lower levels of HDL in comparison with controls [149]. Treatment with dopamine agonists decrease LDL levels [153,154].

### 4.3. Cardiovascular System

Prolactin may act on ventricular myocytes [157]. It exerts a direct inotropic positive action on the mammalian myocardium [158].

Hyperprolactinemia in humans may be associated with a slight impairment of diastolic function [159]. Epicardial adipose tissue thickness (EATT) and carotid IMT (cIMT) are greater in patients with prolactinoma, despite their normal cardiac systolic and diastolic functions [160,161]. Subclinical cardiac dysfunction has been observed in untreated patients and is characterized by impaired LV systolic and diastolic function, as well as regional segment motional abnormality [87]. The prevalence of OSAS in patients with prolactinoma is similar to that in obese subjects and does not change after 6 months of DA treatment. Prolactin levels do not seem to be related with OSAS development [162].

### 4.4. Effects of Specific Treatments on Metabolic and Cardiovascular Issues

Cabergoline improves insulin sensitivity and inflammatory markers and causes a decrease in cIMT independent of the decrease in prolactin, LDL cholesterol and BMI [155]. Rapid decline in prolactin by pituitary surgery improves lipid metabolism, whereas high dose cabergoline (≥2 mg/week) exerts a beneficial impact on both insulin secretion and peripheral sensitivity [163]. During treatment with DAs, monitoring for cardiac valvulopathy is necessary [164,165].

A paradoxical increase of IGF-1 has been noted in patients treated with DA for prolactinomas but whether this is related to insulin sensitivity amelioration remains to be elucidated [166].

## 5. Hypopituitarism

### 5.1. Mortality

Patients with pituitary deficiencies constitute a heterogenous group, due to the variable severity of the disease, ranging from an isolated hormone deficiency to multiple ones. The great interest about CV risk (CVR) and hypopituitarism started in the early 1990’s, when Rosen & Bengtsson [167] published on premature mortality from CV disease in hypopituitary patients. CV morbidity is higher in hypopituitary women [168] and SIRs for myocardial and cerebral infarctions are higher in hypopituitary women with NFAs than men [169]. Young age, both in men and women, has been associated with an increased HR for CVR [170,171]. The cause of hypopituitarism is another significant parameter, as for example, craniopharyngiomas exhibit much higher CV mortality than NFAs [172,173]. Moreover, treatment modalities may contribute to CVR, for example radiation therapy [173] and multiple treatments may cause more often pan-hypopituitarism that is known to augment CVR [173].

A metanalysis of 19,153 hypopituitary adults with a follow-up duration of more than 99,000 person years showed that hypopituitarism was associated with increased mortality (weighted SMR, 1.99; 95% CI, 1.21–2.76). In particular, female hypopituitary adults showed higher SMR compared with males (2.53 vs. 1.71). Mortality rate normalized after GH replacement treatment (GHRT) to levels comparable with the general population (SMR with GH replacement, 1.15; 95% CI, 1.05–1.24 vs. SMR without GH, 2.40; 95% CI, 1.46–3.34). Notably, hypopituitary women showed a lower mortality benefit after GH replacement compared with men (SMR, 1.57; 95% CI, 1.38–1.77 vs. 0.95; 95% CI, 0.85–1.06) [12].

Similarly, another metanalysis showed an increased SMR in the studies without GHRT (1.82, 95% CI 1.19–2.46), but not in those with GHRT (1.05, 95% CI 0.84–1.27). However, the mortality rate remained higher in females than in males without and with GHRT. In the cohorts without GHRT, the cumulative SMR was 2.58 (95% CI 1.58–3.57) in females compared to 1.76 (95% CI 1.21–2.31) in males. In the cohort with GHRT, the SMR was 1.20 (95% CI 0.72–1.68) in females compared to 0.99 (95% CI 0.78–1.20) in males [174].

Through the years, the conventional hormone replacement in hypopituitary patients has been considered far from ideal. It has been gradually understood that the conventional glucocorticoid replacement was actually an over-treatment. Thyroxine replacement tended to be underdosed [175], and low FT4 was associated with increased BMI and Waist-Hip-Ratio (WHR) and low HDL levels. As estrogen is often not substituted, the exact CVR for every hypopituitary patient is quite individualized.

### 5.2. Growth Hormone Deficiency

#### 5.2.1. Body Composition

Adult patients with GH deficiency (AGHD) are often obese and exhibit increased WHR due to excess abdominal body fat. They have low resting energy expenditure and studies have shown that GH treatment can improve body composition [79,176]. The ability of GH to bind in adipose GH receptors and affect the mitochondrial-maintained body temperature [177] is a possible explanation.

GH deficiency (GHD) causes an up-regulation of the enzyme 11β-HSD1, which catalyzes the conversion of cortisone to active cortisol. This cortisol excess contributes to weight gain and body changes, especially when hypopituitary patients also receive hydrocortisone (HC) replacement.

#### 5.2.2. Metabolism

The prevalence of metabolic syndrome in hypopituitary patients is increased by 20–50% compared with the general population [178]. The risk is higher in GH deficient patients -especially females-, those with adult-onset disease and patients older than 40 years [179,180].

GHD is associated with elevated total and LDL-cholesterol and TGs, as well as decreased HDL levels, particularly in females [181,182]. Lower concentrations of apo A1 and higher levels of apolipoprotein B have also been reported [183]. Worsening of lipid profiles along with increased BMI has been reported in young patients who discontinue GH treatment during transition from childhood to adult GH therapy [184]. GH replacement in 12 GH deficient adults did not significantly change their IHL and their insulin resistance HOMA-IR index [79]. A meta-analysis demonstrated that short duration (6–12 months) of GHRT is associated to a deterioration in glucose metabolism including FPG, fasting insulin (FI), HbA1c and HOMA-IR in AGHD patients. These negative effects were not seen in longer duration of GHRT, except for FPG [185].

#### 5.2.3. Cardiovascular System

Atherosclerosis of smaller and larger vessels is a frequent finding in hypopituitary patients [186]. Increased cIMT [187,188] has been detected. Patients with GHD exhibit decreased fibrinolysis, primarily due to increases in PAI-1 [189], fibrinogen and thrombin antithrombin complex [108]. Increased levels of C-reactive protein and pro-inflammatory cytokines (such as IL-6 and TNF-α) [190] together with altered platelet function and subsequent increased adhesion rate [191] contribute to endothelial dysfunction.

GHD can potentially lead to structural and functional cardiac abnormalities, eventually culminating in diastolic dysfunction and myocardial hypokinesia. The LV mass is often reduced and patients may have low cardiac output and ejection fraction and subsequently a compromised exercise capacity. A meta-analysis of cardiac MRI studies has confirmed the ventricular alterations both in the right and left ventricle of these patients [192].

GHD has been detected in roughly 30% of chronic heart failure patients and GHD patients show impaired functional capacity and LV modelling and elevated serum N-terminal pro-brain natriuretic peptide (NT-proBNP) levels in comparison with GH sufficient patients [193]. GH replacement in AGHD patients improves systolic function [194]. In patients with chronic heart failure and GHD, GH replacement improved their peak oxygen consumption (VO_2max_), LV ejection fraction and volumes and their serum NT-proBNP [195].

The effect of long-term GH treatment on CV outcomes has been evaluated in patients with AGHD who received GHRT for up to 10 years as part of the observational multicenter programs NordiNet^®^ IOS in Europe and Middle East and the ANSWER Program in USA. The risk was lower for patients treated with GH for 2- and 7-years vs. age- and sex-matched control groups (14.51% vs. 16.15%; *p* = 0.0105 and 13.53% vs. 16.81%; *p* = 0.0001, respectively) [196].

### 5.3. Secondary Cortisol Deficiency

#### 5.3.1. Body Composition and Metabolism

The effect of secondary cortisol metabolism on body composition and metabolism are intermixed with the effects of other anterior pituitary hormone deficiencies. Over-treatment with supraphysiologic HC doses and inappropriate timing has been associated with increased morbidity, and obesity [197] and CV morbidities as (a) atherosclerosis and increased vessel resistance, (b) systolic and diastolic heart dysfunction and (c) impaired heart contractibility [198]. Sherlock et al. demonstrated significant abnormalities in corticosteroid metabolism in patients with ACTH deficiency treated with conventional doses of HC therapy and in particular increased 11β-HSD1 activity, which is associated with central adiposity [199]. HC equivalent doses of at least 20 mg/d in adults with hypopituitarism are associated with an unfavorable metabolic profile [200]. Novel regimen schemes and GC preparations try to better mimic endogenous cortisol rhythm. Treatment with dual release HC has led to reductions in BMI and waist circumference [201] and a more favourable metabolic profile [202].

#### 5.3.2. Cardiovascular System

Cortisol deficiency affects myocardial contractility, and may cause QTc prolongation, being a causative factor for torsade de pointes, left ventricle hypokinesia and T wave inversion [203]. Glucocorticoids are important for the calcium transport in heart muscle and up-regulate expression of ion channels [204].

Reports on secondary adrenal insufficiency and coagulation are scanty. Patients with Sheehan’s syndrome demonstrate thrombocytopenia, shorter PT and aPTT and von Willenbrand factor deficiency [205].

Higher doses of HC increase systolic and diastolic BP through changes in the renin-angiotensin-aldosterone system, 11β-HSD activity and circulating normetanephrine [206].

Behan et al. compared three HC dose regimens: dose A (20 mg mane and 10 mg tarde), dose B (10 mg mane and 10 mg tarde) and dose C (10 mg mane and 5 mg tarde) in hypopituitary men. There were no differences in 24 h BP between dose regimens and controls; however, low-dose HC replacement (dose C) was associated with the lowest arterial stiffness index compared with the other dose regimens and maintenance of nocturnal blood pressure dipping [207].

### 5.4. Gonadotrophin Deficiency

#### 5.4.1. Metabolism

Hypogonadism is frequently untreated in hypopituitary patients, as only 23.3% of male patients under 60 and 24.4% of female patients under 50 receive gonadal replacement therapy.

Gonadal steroids affect body composition and intermediate metabolism. Low testosterone increases body fat mass and is associated with insulin resistance, metabolic syndrome, type II diabetes and obesity [208]. The lipid profile associated with low testosterone is atherogenic, with increased total, LDL cholesterol and TGs [209] and low HDL cholesterol and apo A1 [210]. Testosterone replacement increases plasma hepatic lipase activity and causes small increases in small dense LDL cholesterol [211].

Low estradiol levels in menopause and hypogonadism are associated with an increase of total and central body fat and reduced insulin sensitivity. Elevated LDL cholesterol occurs frequently, although the lipid derangements of the metabolic syndrome are also observed [212].

#### 5.4.2. Cardiovascular System

Hypogonadal untreated patients have increased mortality [172] due to CV morbidities. Low testosterone correlates with heart failure severity. Nevertheless, some data on testosterone replacement in hypogonadal men also demonstrated an increase in CV events [213].

Cardioprotection in women is lost in menopause as estrogens deplete and diastolic dysfunction, cardiac hypertrophy, ventricular stiffness and heart failure may appear. Studies on women with premature menopause show an increased risk of nonfatal CV disease, including coronary heart disease and stroke, events that occurred before the age of 60 [214]. In this context, hormone replacement therapy probably gives a benefit in younger patients and after a 5-year period of treatment [215]. Based on the Women’s Health Initiative trials, a risk-benefit approach is also preferable in hypopituitary hypogonadal female patients.

### 5.5. Thyrotropin Deficiency

#### 5.5.1. Metabolism

A complex interplay exists between thyroid hormones and body composition, glucose and lipid metabolism. Hypothyroidism has been associated with insulin resistance and current studies indicate a higher risk for type II diabetes in these patients [216].

Central hypothyroidism is usually treated, as it is easy to detect and initiate therapy. However, we anticipate in some cases mild derangements in thyroid hormone levels. Patient profiling in hypothyroidism shows increases in total, LDL cholesterol, TGs and Lp(a). The reduction of cholesterol uptake due to down-regulation of hepatic LDL-receptors is a significant pathway in hypothyroidism. In addition, as thyroid hormones induce HMG CoA reductase activity and enzymes for lipoprotein metabolism and reductions in apo-B48 and apo-B11 [217], lipid abnormalities in hypothyroidism may be attributed to these additional mechanisms.

#### 5.5.2. Cardiovascular System

Some patients, especially those with older age and with co-morbidities, may be under-treated. In addition, despite the normal peripheral hormone levels, local cardiac hypothyroidism may add to the adverse effects on heart, due to upregulation of type 3 iodothyronine deiodinase [218].

As hypothyroidism may cause systolic and diastolic dysfunction, heart failure may ensue in these patients. Heart failure with preserved ejection fraction has been observed in patients with overt and subclinical hypothyroidism and has been associated with CV mortality [219]. A metanalysis estimated that although LVEF was lower in patients with hypothyroidism than in healthy people levothyroxine replacement therapy reverses cardiac function [220].

The renin-angiotensin-aldosterone system is downregulated in hypothyroidism. A similar downregulation has been reported for the beta-adrenergic system in cardiomyocytes. Therefore, in untreated hypothyroidism bradycardia, reduced heart contractibility, narrowed pulse pressure and low cardiac output may be observed.

**Table 2:** CV risk factors in pituitary disorders: recent findings.

Cushing	Acromegaly	Hypopituitarism
-Mortality is increased in patients with active disease and those in remission and CVD is the main cause [13] -Chronic hypercortisolaemia is associated with cardiovascular abnormalities, which do not reverse completely even after remission of CD [2,3,4,5,6,7,8,9,17,22,24,36]	-Mortality is decreased, but CVD is still the 2nd cause of death in acromegaly [62] -Increased CV risk is due to the presence of several pro-atherogenic factors and comorbidities [100] -Hypertension is still the most important factor for increased mortality and should be treated early and rigorously [86] -Endothelial dysfunction and systemic inflammation are key players and persist in treated patients [89] -New cardiovascular markers are evaluated in acromegaly [114] -Overtreatment of acromegaly should be avoided [146]	-Female hypopituitary adults show higher SMR compared with males [12]. -Mortality rate normalizes after GH replacement in levels comparable to general population but females have a lower mortality benefit after GH replacement compared with men [12]. -Cardiovascular morbidity is higher in females with hypopituitarism [168,169]

## 6. Conclusions

Modern therapeutic armamentarium has reduced total mortality of pituitary disorders but CV morbidity remains the main burden.

In acromegaly mortality is on a decreasing slope, but CVD is still a significant contributor, ranking second after malignancies. This is linked to the co-existence of several pro-atherogenic factors and comorbidities. Recent research focuses on endothelial dysfunction and systemic inflammation, while new circulating markers are being investigated. Certain CV alterations are only partially reversible with treatment and should be checked and treated early in the course of acromegaly.

Mortality in patients with CD is remarkably eleveated in the active phase and remains still high but in lower level in patients in remission demonstrating the dramatic and irreversible effect of hypercortisolaemia. Cortisol excess leads to significant metabolic derangements and unfavorable heart remodelling with subsequent elevated CV morbidity. Patients with prolactinomas suffer very often from obesity and insulin resistance. Mortality is increased in hypopituitary patients especially in females and young patients and CVD is the main contributor. GH deficiency and chronic glucocorticoid replacement are associated with metabolic syndrome and increased oxidative stress. Mortality rate in hypopituitary patients normalizes after GH replacement in levels comparable to general population although female patients benefit less than males.

Early diagnosis and successful management of pituitary disorders is challenging and the only pathway to normalize the CV burden of pituitary diseases.

## Figures and Tables

**Table 1:** Metabolic and cardiac structural and functional alterations in patients with GH deficiency, acromegaly, prolactinoma and CD.

	GHD	Acromegaly	Prolactinoma	Cushing’s Disease
Lipid metabolism	Increased total cholesterol & LDL Increased triglycerides Decreased HDL	Increased Lp(a) Increased triglycerides Decreased HDL	Increased LDL Decreased HDL	Increased total cholesterol & LDL Increased triglycerides Decreased HDL
Insulin resistance/ metabolic syndrome	Obesity Increased subcutaneous and visceral fat Glucose intolerance	Reduced beta cell function Increased lipolysis Decreased glucose uptake Increased glycogenolysis & glyconeogenesis	Obesity Insulin resistance	Obesity Insulin resistance Diabetes mellitus
Endothelium and vasculature	Increased carotid intima media thickness Atherosclerosis of small and large vessels Reduced ascending aorta diameter	Endothelial dysfunction Increased endothelial proliferation Increased oxidative stress		Increased intima-media thickness of carotid and aorta Atherosclerotic plaques
Coagulation	Increased PAI-1 Reduced protein S	Increased fibrinogen and PAI-1 Decreased t-PA and TFPI		Hypercoagulability
Pro-inflammatory markers and oxidative stress	Increased c-reactive protein Increased adipokines & pro-inflammatory cytokines (IL-6 and TNF-α) Increased PAPP-A Elevated oxidated LDL	Increased proinflammatory cytokines Overexpression of cell adhesion molecules		Increased proinflammatory cytokines
Cardiac structure and function	Diastolic dysfunction Myocardial hypokinesia Reduced left cardiac ventricular mass Low ejection fraction Compromised exercise capacity Hypertension	Left ventricular hypertrophy Systolic & diastolic dysfunction Cardiomyopathy Valvulopathies Hypertension	Subclinical cardiac dysfunction	Left ventricular hypertrophy Systolic & diastolic dysfunction

Abbreviations: low-density lipoprotein (LDL), high-density lipoprotein (HDL), left ventricle hypertrophy (LVH), plasminogen activator inhibitor (PAI-1), pregnancy associated plasma protein A (PAPP-A), tissue plasminogen activator (t-PA), tissue factor pathway inhibitor (TFPI).
